# New Sustainable Solvent Extraction Pathways for Rare Earth Metals via Oximes Molecules

**DOI:** 10.3390/molecules28227467

**Published:** 2023-11-07

**Authors:** Maria Atanassova, Rositsa Kukeva, Vanya Kurteva

**Affiliations:** 1 Department of General and Inorganic Chemistry, University of Chemical Technology and Metallurgy, 8 Kliment Okhridski Blvd., 1756 Sofia, Bulgaria; 2 Institute of General and Inorganic Chemistry, Bulgarian Academy of Sciences, Acad. G. Bonchev Street, Block 11, 1113 Sofia, Bulgaria; 3 Institute of Organic Chemistry with Centre of Phytochemistry, Bulgarian Academy of Sciences, Acad. G. Bonchev Street, Block 9, 1113 Sofia, Bulgaria

**Keywords:** oximes, extraction, synergism, lanthanoids, ionic liquids, selectivity, EPR

## Abstract

A study on the synergistic extraction of Eu(III) ions with a series of chelating ligands and determination of the process parameters is presented by employing ionic liquids and typical organic diluents. The investigations of the liquid–liquid extraction, commonly applied in the separation science of 4f and 5f-ions acidic chelating compounds, 4-benzoyl-3-methyl-1-phenyl-2-pyrazolin-5-one (HP), 4-benzoyl-3-phenyl-5-isoxazolone (HPBI), and 2-thenoyltrifluoroacetone (HTTA) alone and in combination with two synergistic agents, *meso*-hexamethylpropyleneamine oxime (S2: HM-PAO) and its bis-imine precursor (S1: pre-HM-PAO), are presented. The interaction between the two extractants (acidic/neutral) in deuterochloroform was studied using ^1^H, ^13^C, and ^1^H-^1^H NOESY experiments. Several conclusions are given highlighting the role of the ionic diluent in complexation processes and selectivity with an employment of the two synergistic agents for various metal s-, p-, d-, and f-cations in the Periodic table, with almost 25 metal ions. The objective was to optimize a system for 4f-ions solvent extraction based on the new oxime molecules with β-diketone/isoxazolone/pyrazolone partnership. As detailed above, slight enhancements of extraction efficiencies were obtained either by using basic synergistic agents such as HM-PAO and/or using pre-HM-PAO. A competitive solvent extraction test of nearly 18 f-ions by various ligands (HTTA, S1, S2, and HPBI) and the two mixtures HTTA−S1 and HTTA−S2 diluted in ILs or organic diluents was also conducted in order to evaluate the switchable diluent impact. Additionally, electron paramagnetic resonance (EPR) spectroscopy was used to study the established chemical species with Cu^2+^ cations in the obtained organic extracts involving the two synergistic molecules.

## 1. Introduction

For a long time rare earth metals remained laboratory curiosities only, although Carl Auer von Welsbach (1858–1929, world-famous at 27 years) initiated some applications in lighting, as he took patents out for the famous Auer mantle for gas lamps (1891) and for flint stones (1903), and founded two companies (1898) that in some way are still active today [1]. There is no doubt that the inherent chemical and physical properties of these metals and their compounds have been understood more quantitatively with the improvement of research tools and chemical technologies [2,3,4]. Beyond that point, the investigation of molecular coordination complexes of 4f-ions has attracted significant attention due to their outstanding properties [5,6], considering the growing levels of lanthanoids present in the technosphere today and their numerous applications [7,8,9]. The coordination chemistry of lanthanoids in conjunction with modern analytical chemistry plays an important role in the development of innovative compounds for technological, industrial, and biological applications [10,11].

Over the past decades, hundreds of organic ligands with various structures and high selectivity relative to groups of ions or individual types of ions have been obtained by researchers. The organic chemists have rendered yeoman’s service by synthesizing an array of efficient ligands with different functional groups containing various donor atoms (P, S, N, etc.) [12,13,14,15,16,17,18,19,20]. As an example, a di-quinolinol chelating ligand 2,2′-((octylazanediyl)bis(methylene))bis(quinolin-8-ol) was synthesized recently for the selective extraction of Mo^6+^ by an ionic liquid system (diluted in [C_1_C_4_im^+^][Tf_2_N^−^]: (cations are denoted as [C**_n_**C**_m_**im^+^] for *n*-alkyl-*m*-alkylimidazolium, whereas the anions are denoted as [(CF_3_SO_2_)_2_N^−^] (bis(trifluoromethylsulfonyl) imide, [Tf_2_N^−^])) from the acid nitric solutions of uranium targets containing U^6+^, Cs^+^, Ru^3+^, Ce^3+^, Te^4+^, and I^−^ [21]. Ueda et al. reported the platinum group separation of precious metals from base metals (Fe^3+^, Co^2+^, Ni^2+^, Cu^2+^, and Zn^2+^) achieved by the phenylurea derivative of a trident molecule in chloroform [22]. The authors explained the observed outstanding difference in selectivity with the fact that the base metals exist as cationic species in nitrate media, so counter anions are necessary for charged neutralization (M^n+^) and further extraction by neutral ligand. In an ionic liquid environment, the NH-urea containing ring molecules exhibited distinctive selectivity in comparison with CHCl_3_, in which the diluent extraction percentage was below 5%, independent of the nitric acid concentration (0.001 to 8 mol/dm^3^). Further, in general, the tetradentate diiminedioxime ligands behaved like dioxime or aminooximes by enveloping themselves around the metal ions in a planar geometry, forming a hydrogen bond between the two oxime groups by removing one hydrogen ion [23,24,25]. As a whole, lanthanoid ions preferred to bind to hard donors such as O and F, rather than to soft bases, such as P and S donor ligands [26,27]. On the other hand, nitrogen-containing ligands were an apparent anomaly, since they could form relatively few complexes, at least partly due to the high basicity of some specific molecules. For instance, by 1964, the complexation of the lanthanoids in a solution was studied for only 16 organic ligands with a sufficient number of cations, in order to establish the trends in the stability constants across the 4f-series [28]. Liquid–liquid extraction is a key process in metal refining, and it is the only industrial technology for the intra-group separation of rare earth elements [11,12,19,29]. Moreover, complex formation is pH dependent, and the stabilities of the chelates are related to the ionic radii of the rare earth ion as well. Purely on steric grounds, therefore, it would be expected that 4f ions could accommodate more than six ligands in their coordination sphere [30]. However, the search for new organic molecules that exhibit the ability to form complexes continues. The main reason for such a hunt is the increasing risks of environmental pollution with increasingly diverse ecotoxicants, and the presence of various metal ions together in one matrix is a serious difficulty to separate [31,32,33]. Metals are infinitely recyclable in principle, but in practice, they are often inefficient or essentially nonexistent for some of them; i.e., currently, we are far from a closed-loop waste management [34,35,36,37].

This research investigation presents the synthesis and complexation ability of two oxime molecules used as synergistic agents during liquid–liquid extraction of Eu^3+^ ions in combination with three chelating ligands as well as used alone for various metal ions with different charges from the Periodic table. In order to present the details of this study, the Eu^3+^ ion (congener of Am) was selected as a representative of the middle of the 4f-series in view of an indirect comparison study due to the difficulties in accessing and handling actinoids. Due to the same oxidation state and very similar ionic radii, the chemical properties of 4f and 5f ions are very similar; as a consequence, their mutual separation is considered to be one of the most challenging issues. To the best of our knowledge, so far, no such detailed correlations, as in the case of the chemistry of the chelating ligands, have been established for the oxime-bridged molecules, and there is a need to collect more experimental data. To advance the understanding of the oxime-based separation processes, it is necessary to control binding via various metal ions. It will be better to understand the contribution of the acidic and basic extractants to synergism as a phenomenon and to obtain enhanced selectivity in different organic mediums without looking at the molecular or ionic mechanism aspects. The main focus is of course the ability to achieve good selectivity in the 4f series by applying different solvent systems. The other aim is to study the possible influence of the interaction between the two extractants on the synergistic process plus the introduction of EPR analysis in extraction chemistry.

## 2. Results and Discussion

### 2.1. NMR Interactions Study between the HPBI Compound and the Two Synergistic Agents

**Figure 1:**
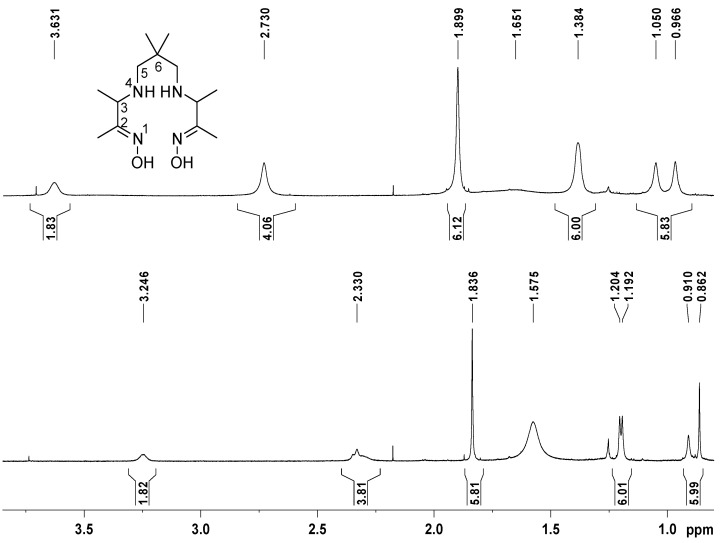
HM-PAO signals in the ^1^H NMR spectra of HM-PAO (**bottom**) and HPBI:HM-PAO, 1:1 mixture (**top**) mixture.

The probable interaction between the two extractants usually applied in the synergistic solvent extraction of metals is an essential problem that cannot be neglected [38,39,40]. The debates about the interaction between the extractants in mixed systems have arisen almost immediately with the appearance of the first studies devoted to the synergistic solvent extraction of metal ions. Firstly, Marcus and Kertes [41] noted that a direct interaction between the extractants has an appreciable effect on the breakdown of synergism, but later studies have shown that both phenomena (synergism and antagonism) are much more complex [42,43]. At this point, based on the literature review, it may be clear that the possible interaction of ligands during the extraction of metallic species by mixtures of two or more compounds is not a reaction with foremost importance when synergism occurs [37,38]. As a whole, relatively few studies were devoted in the past to the destruction of synergism, and even fewer papers have been published on this subject in recent years. These research studies are important for elucidating the behavior of different combinations of extractants in the organic phase. In the literature, the latter is referred to as critical for achieving synergistic effects. The possible reactions between ligands, the strongest chelation (HPBI) chosen for this investigation, were examined by NMR technique. The research task was to obtain new data on ligand-ligand interactions, as well as to evaluate the influence of the chemical structure of the extractants by introducing different functional groups in the compounds. The NMR spectra of the main extractant molecule, the two synergistic compounds, and their 1:1 mixtures were recorded in chloroform-d. Significant chemical shift changes in the signals in the proton spectra of both synergistic agents were observed upon mixing, as illustrated for the HM-PAO compound (S2) in Figure 1.

**Figure 2:**
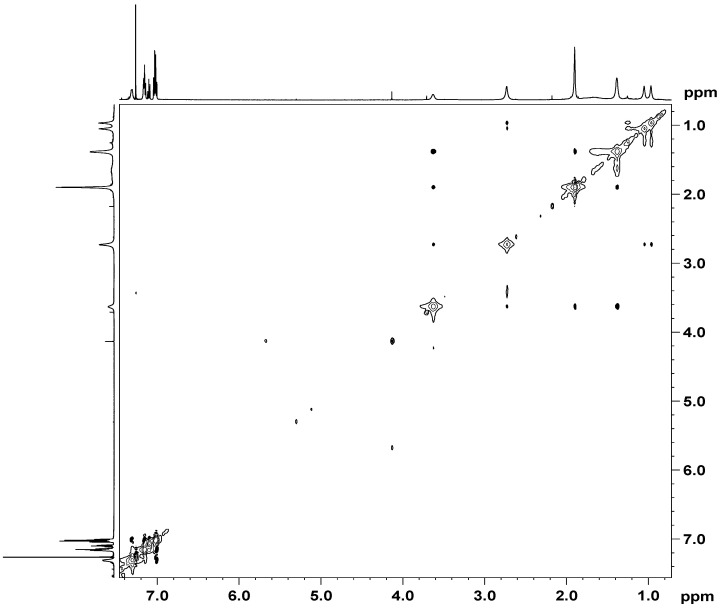
^1^H-^1^H NOESY spectrum of HPBI:HM-PAO, 1:1 mixture.

As can be seen, all HM-PAO signals, except for CH_3_-3, are shifted downfield in the mixture. The effect is the most substantial for the CH-3 and CH_2_-5 signals, which is an indication that NH-4 groups are involved in interactions with the isoxazolone molecule. However, it is clear that the signals for one of the isoxazolone phenyl groups in the mixtures with both synergists are significantly shifted up-field, whereas those for another possess almost the same chemical shifts (see Figure S1 in the Supporting Material). These observations are consistent with the interactions between the isoxazolone carbonyl function and HM-PAO amino groups. This suggestion is confirmed by the NOESY experiment, where only intramolecular interactions are registered (Figure 2); i.e., the synergists are located on the OH…C=O isoxazolone area with phenyl groups on the opposite side.

### 2.2. Investigation of Synergism in IL Medium or CHCl_3_ Implementing Three Chelating Ligands (HL) in Combination with Two Oximes, i.e., HM-PAO or Pre-HM-PAO

The efficient transfer of metal cations from the upper aqueous phase into a lower usually organic medium is most readily achieved via conventional extractants capable of satisfying, to some extent, their coordination and solvation requirements, or entirely if possible [44]. The main reason for synergism as a phenomenon in molecular diluents is due to the occurrence of a new mixed species containing both ligands: one to neutralize the charge of the metal ion via the formation of a chelate complex for example, whereas the second molecule expels any residual coordinated water, thereby yielding a more organophilic metal complex [45]. When the cation partitioning is greater for the combined extractants than the sum of their individual employment, the result is estimated as a synergistic effect. However, this picture is much more complicated and incomplete because the ionic character of the organic phase offers a different set of design criteria for metal coordination in solution [44,46,47,48,49]. Undoubtedly, the acidic strength of the chelating ligand and the pH of the aqueous phase are also important in ionic extraction systems [50,51] despite the fact that until now, the main focus has been on the combination of two neutral extractants as well as the acidic–neutral ligand duo [37]. In this context, additional investigation was undertaken in the present work in order to establish a certain relationship between the mechanism and coordination state of lanthanoid complexation in an ionic liquid environment with an acidic–basic mixture of ligands, which is rarely studied. The experimental data showed that 4f-ions extraction with the two synergistic compounds alone is negligible under the experimental conditions of the present study: 1 mM applied concentration and pH range 1.11–5.6 in the presence of buffer. Unfortunately, as anticipated, taking into consideration the achieved coordination number (C.N.) in the HPBI/[C_1_C_4_im^+^][Tf_2_N^−^] system, the extraction of lanthanoids with the two outline mixtures is not appreciable, as shown in Figure S2. The values of *D*_Ln_ observed at a given pH are almost identical to those calculated on the basis of individual chelating ligand contributions of HPBI, Figure S3. Therefore, the synergistic effects of such systems are rather nil for lanthanoids. This situation is not a failure of the framework but rather reflects the complexity of similar ionic-based systems when the strongest, [51] moderate, or little enhancement has been produced upon the addition of a second ligand [46,50,52,53], as well as when the anti-synergistic phenomenon was detected for heavy rare earth elements only (from Gd to Lu) [54]. One might be asked why synergistic enhancement does not occur in this case albeit similar situations exist in molecular liquids. The analogy is imperfect, taking into account that the degree of coordination number of 4f-ions is already high in a single ligand system and the geometry of the second compound is not so favorable for bonding lanthanoids. In many cases, it is very difficult to predict the adduct-forming tendency of a donor molecule. Taken together, these results strongly indicate that decomposing the stable species La(PBI)_3_·HPBI and [Ln(PBI)_4_]^−^ in a new mixed ligand arrangement is not an easy-going reaction in an ionic ambiance, for example. The four anions of the chelating molecule (PBI^−^) form the inner coordination sphere of the complex, satisfying the coordination abilities of lanthanoid ions (C.N. = 8). In contrast, in molecular liquids, the formation of mixed adducts of the type LnL_3_·S incorporating neutral organophosphorus derivatives [51,52] or second β-diketone [54] is a plausible equilibrium process, which is much more favorable than the self-synergism LnL_3_·HL [37]. The balance between diverse mechanisms in the IL surrounding is uncontestably absolutely complicated, but there appear to be certain tactics by which it can be influenced in the preferred direction in order to accomplish the desired objectives [55].

**Figure 3:**
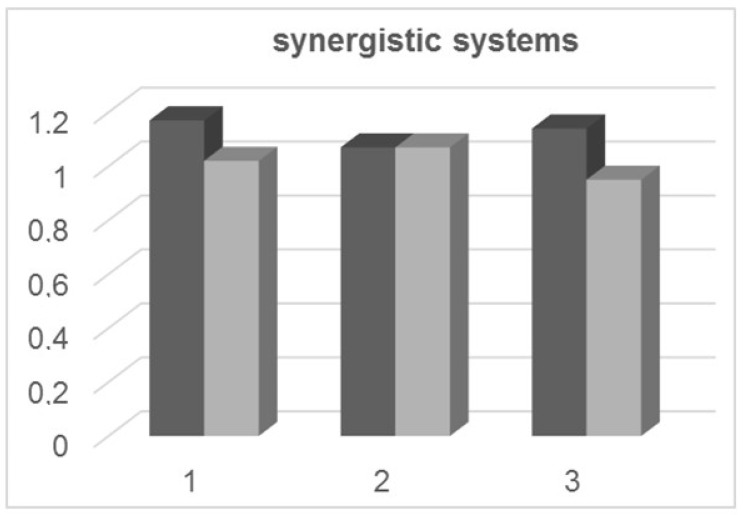
Log*D*_L,S_ for Eu^3+^ ([Eu^3+^]_in_ = 2.5 × 10^−4^ mol/dm^3^, 0.1 mol/dm^3^ NaCl, pH_in_ = 2.50 (HCl), V_aq_:V_o_ = 2:1 mL (CHCl_3_)) at: 1. [HPBI] = 8 × 10^−3^ + [S1] = 6.7 × 10^−3^ mol/dm^3^ (pH_eq_ = 7.08) (black) and [HPBI] = 8 × 10^−3^ + [S2] = 5 × 10^−3^ mol/dm^3^ (pH_eq_ = 2.81) (grey); 2. [HP] = 1 × 10^−2^ + [S1] = 6.7 × 10^−3^ mol/dm^3^ (pH_eq_ = 6.50) (black) and [HP] = 1 × 10^−2^ + [S2] = 5 × 10^−3^ mol/dm^3^ (pH_eq_ = 4.04) (grey); 3. [HTTA] = 2 × 10^−2^ + [S1] = 5 × 10^−3^ mol/dm^3^ (pH_eq_ = 6.57) (black) and [HTTA] = 2 × 10^−2^ + [S2] = 5 × 10^−3^ mol/dm^3^ (pH_eq_ = 5.26) (grey).

Three different combinations of ligands involving different chelating molecules and two oximes were tested to induce a further increase in the extraction of the lanthanoid series metals in CHCl_3_. The obtained results for europium chosen as a representative of the 4f-series are presented in Figure 3. The overall superiority of the S1 over the S2 ligand is slight under the relatively invariable conditions of the experiments.

The chemical structures of the two synergists, which lead to a significant increase in the pH of the aqueous phase during the process, cannot be ignored, as shown in Figure S4. In the presence of chelating acid ligands, the effect seems to be less significant. However, in their absence, i.e., when the two oxime molecules act alone upon establishing a difference between the initial pH and after a certain period of time, a substantial rise in the pH value to approximately 9 could be detected. This observation indicates a marked disadvantage in relation to lanthanoids, which are usually extracted under relatively more acidic conditions in an aqueous environment.

**Figure 4:**
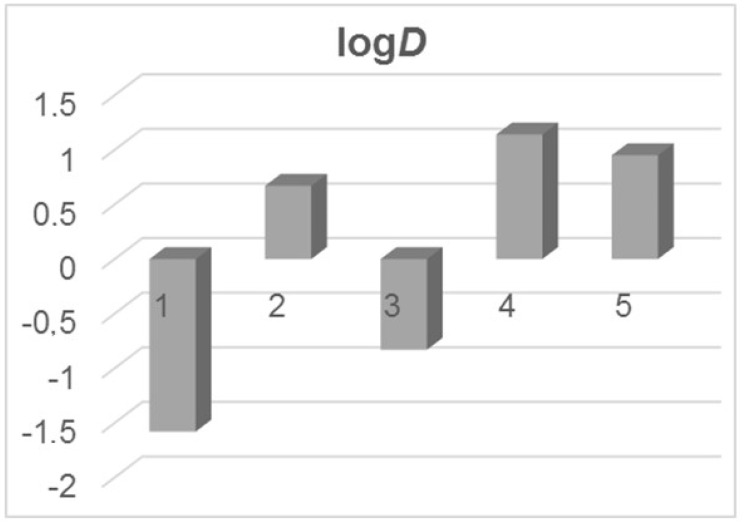
Log*D* for Eu^3+^ at: 1. [HTTA] = 8 × 10^−2^ mol/dm^3^ (pH_eq_ = 2.51); 2. [S1] = 5.2 × 10^−3^ mol/dm^3^ (pH_eq_ = 7.56); 3. [S2] = 4.04 × 10^−3^ mol/dm^3^ (pH_eq_ = 6.06); 4. [HTTA] = 2 × 10^−2^ mol/dm^3^ + [S1] = 5 × 10^−3^ mol/dm^3^; 5. [HTTA] = 2 × 10^−2^ mol/dm^3^ + [S2] = 5 × 10^−3^ mol/dm^3^.

Further, the influence of the introduction of a second extractant into the HTTA system in the form of a synergist, S1 or S2, on europium extraction was investigated in CHCl_3_. An expected positive effect was obtained in this case [47,51], as an increase can be seen in the results presented in Figure 4 It should be noted that the concentration of the main extractant in this case is of decisive importance. Unfortunately, almost no extraction was observed at rather high concentrations of HTTA as 2 × 10^−2^ mol/dm^3^, 3.5 × 10^−2^ mol/dm^3^, or 5 × 10^−2^ mol/dm^3^ when it is used alone. At the same time in a synergistic combination, this concentration is drastically reduced (Figure 4).

The synergistic enhancement upon the addition of a second extractant can be assessed using synergistic coefficients calculated as SC = log (*D*_1,2_/*D*_1_ + *D*_2_), where *D*_1,2_, *D*_1_, and *D*_2_ denote the distribution ratios of a metal ion using a mixture of extractants (*D*_1,2_) and the same extractants separately (*D*_1_ and *D*_2_). Therefore, the required calculations were performed for the nine investigated solvent systems, and the obtained values are presented in Table 1. It is estimated that the two systems that show little ability to extract europium like HTTA+S1/S2 have a greater synergistic coefficient. S2 produces a more significant effect on the Eu^3+^ extraction, and the combination HTTA+S2 highly enhances it. Of course, if a strong powerful ligand such as HPBI was used, there is no need to introduce a second molecule into the solvent system; therefore, in this study, a negative value was obtained. Hence, the observed deviation concerning synergism is probably due to the rather large difference in the p*K*_a_ values of the three chelating molecules [54]. However, the addition of phosphorus containing calix[n]arenes to the chelating ligand improves the efficiency of Ln^3+^ ion extraction and produces large synergistic effects of up to 10^3^ [52]. The latter is a result of the tunable shape and flexibility of the unique three-dimensional structure of these synergistic molecules. Moreover, compounds possessing P=O groups like phosphates, phosphines, and phosphine oxide derivatives, have shown high affinities for f-elements [46,51].

Unfortunately, no extraction of Eu^3+^ ions was detected at [HTTA] = 8 × 10^−3^ mol/dm^3^, varying the concentration of the compound S2 dissolved in CHCl_3_ at lower pH values (pH_eq_ = 3.50−2.15), i.e., in the range of 1 × 10^−3^ to 6 × 10^−3^ mol/dm^3^. Therefore, the dependence on the pH of the aqueous medium is of paramount importance, and a fairly high concentration of this chelating compound is required in the extraction process using molecular diluents such as chloroform, which is a serious drawback (Figure S5).

### 2.3. Solvent Extraction and Selectivity across the Periodic Table and 4f-Series

**Figure 5:**
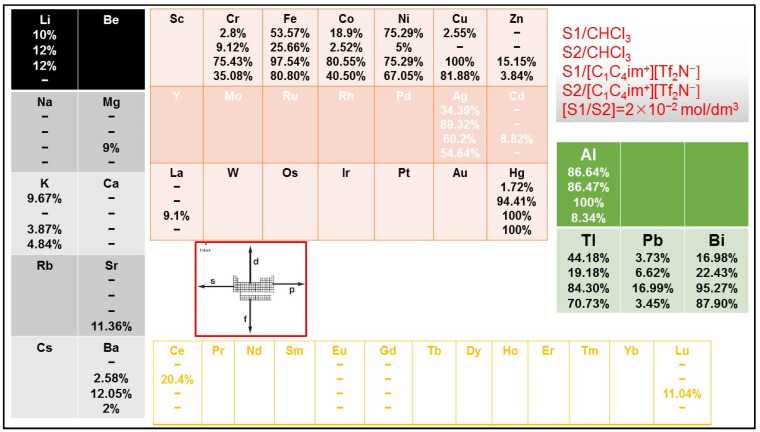
Extraction performance of S1 and S2 ligands towards 25 metal ions. The reported extractability values (%) represent the average of three measurements, with deviations of less than 5%.

From the research study aimed at evaluating the solvent extraction ability of the two synthesized oxime molecules towards various metal cations, it can be seen that they are not so suitable for lanthanoids, as shown in Figure 5. This cannot be explained by the cation charge +3 or radius because the chemical element bismuth is extracted extremely well with both ligands when the medium is an ionic liquid. Secondly, we can note the exceptional influence of the diluent used in the solvent system. Since it is an ionic liquid ([C_1_C_4_im^+^][Tf_2_N^−^]), the extraction is much more efficient compared to the molecular diluent, i.e., chloroform, regardless of the metal belonging to the s, p, d-, or f- block. However, almost the same efficiency is demonstrated for the Hg^2+^−S2 and Ni^2+^−S1 systems, regardless of the chemical nature of the diluent. The third conclusion that can be summarized is that the S1 molecule seems to be more effective than oxime S2 in most cases, for example, cations such as Cr^3+^, Fe^3+^, Co^2+^, Zn^2+^, Al^3+^, Pb^2+^, and Ba^2+^. In general, both oxime molecules can also be successfully used for silver solvent extraction.

In order to achieve the sustainability of IL-based solvent systems via the application of new ligands, the selective separation of multi-metal mixtures should be evaluated, especially the behavior of d-f metal ions. This is because of the scarcity of rare earth elements; as a consequence, their recovery from various sources is very important. For example, typical SmCo magnets contain mainly Sm and Co in conjunction with Cu, Fe, Ni, and Zn, which are usually introduced to improve their performance characteristics. In terms of the SFs, the calculated values reported by Deng et al. [56] for Sm/Co and Sm/Cu are 3078 and 54.7 at pH = 6.0, thus providing excellent selectivity during the liquid–liquid mass transfer due to the novel benzyltributylammonium decanedioate IL compound. In the case of the selective separation of lanthanoids and transition metals, Figure 5 shows that the S1/CHCl_3_ and S2/CHCl_3_ systems also exhibit a remarkable separation performance, especially for the coextraction of Ln/Co, Ln/Cu, Ln/Fe, and Cu/Fe pairs. Further, three novel morpholinecarboxylic acid ILs were synthesized for the solvent extraction of Sm^3+^, among which [(CH_2_)_7_COOHmor][Tf_2_N^−^] shows the highest extraction performance [57]. In other words, the extraction efficiency reached ~100% at an initial pH of 1.8 with an extraction equilibrium within 1 min. Additionally, as an associated element, Al^3+^ has a negative effect on the purity of Gd^3+^, due to which three pyridinium-based ILs ([(CH_2_)_n_COOHpyr][Tf_2_N^−^], *n* = 3, 5, 7) were proposed as diluents in the liquid–liquid extraction process for the separation of aluminum impurities from Gd^3+^ by Hu et al. [58]. Thus, in this study, three of the proposed solvent systems are extremely suitable, except for S2/[C_1_C_4_im^+^][Tf_2_N^−^]. Further, solvent extraction of Nd^3+^ and Ni^2+^ ions with the synthesized bi-functional IL based on Aliquat 336 and Cyanex 572 [AL336][Cy572] in kerosene was investigated by Emam and El-Hefny, leading to improved extraction and separation of the two ions compared to the Cyanex 572 extractant used alone [59]. In fact, the highest SF of the Nd/Ni pair of 26.3 was obtained at 0.2 mol/dm^3^ HCl. The research describes a novel method for recycling a real scrap NdFeNi magnet from computer hard disks in order to recover Nd(III) as a marketable salt and other valuable by-products while also establishing an environmentally friendly process. On the other hand, alkyl esters (C_3_–C_8_) of salicylic acid together with trioctylphosphine oxide were considered by Bezdomnikov et al. in order to find new lithium-selective extraction agents because generally, the range of lithium-selective ligands is limited [60]. It was found that the extractability of metals decreases in the series Li ≫ Na > K. It could be argued that the two current ligands under study, S1 and S2, are not so suitable for separating the s-elements in group 1 of the Periodic table, but could be used to separate them from d-ions in a mixture, for example.

In the next scientific study, a competitive solvent extraction investigation of almost all rare earths and two 5f-ions was performed with the chelating ligand HTTA alone or two oxime molecules S1 and S2, as well as in synergistic combinations with CHCl_3_ and IL [C_1_C_6_im^+^][Tf_2_N^−^] at pH < 0.10 of the aqueous phase based on the obtained results shown in Table S1. Unfortunately, under these experimental conditions, these molecules and the corresponding synergistic mixtures cannot be evaluated, as can be seen from the obtained data with 0 % extraction shown in Table S1. Therefore, this necessitated paying special attention to the stronger chelating molecule, where synergism could not be found as a phenomenon in the extraction process, given the data noted above. As a subsequent stage in the present study, the solvent extraction of 18 f-ions was carried out with the chelating extractant HPBI used alone but in different mediums, i.e., nine diluents were tested. This ligand is known in the literature for its effectiveness in strongly acidic media [37,45]. The obtained results are presented in Table 2. It is apparent that this ligand can be used successfully for the separation of Sc and Y, regardless of the organic diluent used. But, the most distinct differences are likely obtained with molecular compounds like CHCl_3_, C_6_H_6_, and C_2_H_4_Cl_2_. And, as a continuation, the impressive influence of the CCl_4_ diluent appears, which is even better compared to the five ionic liquids and cannot be missed in the discussion. It turns out that dichloroethane is highly unsuitable for lanthanoid extraction when combined with this chelate extractant. But, at the same time, it could be used to separate Sc and Th from all the other f elements present in the mixture, which could be useful in practice. Furthermore, the last mentioned chemical element (Th) was extracted above 73% with the nine solvent systems tested regardless of the diluent’s chemical nature—ionic character or molecular, polar, or non-polar diluent. In fact, thorium has received widespread attention as a potential nuclear fuel alternative, but it is still a difficult research task to extract thorium from strong HNO_3_ media [61].

One cannot fail to note the lack of solvent extraction for the light lanthanoids, La, Ce, and Pr, which automatically means that the result, albeit negative, could be useful to some extent in separation chemistry and technology in order to separate them from the heavier representatives in the 4f series. However, it should not be forgotten that they are usually extracted at a higher pH in the aqueous phase, which explains the observed picture. The superiority of ionic liquids is confirmed once again with the scientific data obtained herein, as shown in Table 2. In fact, ionic liquid cations significantly affect their own hydrophobicity and viscosity, which somehow automatically affect the metal cation transfer during the liquid–liquid extraction process [37]. Moreover, when comparing the obtained data for an ionic medium, it can be seen that the efficiency of the extraction process increases in the following order: [C_1_C_4_pyr^+^] < [C_1_C_4_im^+^] < [C_1_C_6_im^+^] < [C_1_C_8_im^+^] < [C_1_C_10_im^+^], as a function of **n**, i.e., with a trend following that of the hydrophobicity of the IL cations. Due to this quantitative observation, the assumption that this molecule would extract lanthanoids in the IL medium more or less similarly, in contrast to the large differences found in molecular solvent extraction systems, seems to be somehow veritable [62,63]. On the other hand, the influence of organic molecular diluents on the extraction process under the applied conditions can be summarized in the order of C_2_H_4_Cl_2_ < CHCl_3_ < C_6_H_6_ < CCl_4_. For instance, benzene has been studied for comparison purposes as a good non-polar diluent, not forgetting that it is currently out of use due to its toxic properties. It should probably be mentioned again that the best results were obtained using the HPBI−CCl_4_ system for all 18 metals. Furthermore, the liquid–liquid extraction of tetravalent zirconium and hafnium from acidic chloride solutions has been investigated using 3-phenyl-4-acyl-5-isoxazolones in xylene, such as HPBI, 3-phenyl-4-(4-fluorobenzoyl)-5-isoxazolone (HFBPI), and 3-phenyl-4-(4-toluoyl)-5-isoxazolone (HTPI), and the following orders were established: HPBI > HTPI > HFBPI for Zr^4+^ and HFBPI > HPBI > HTPI for Hf^4+^ [64]. The effect of the nature of the diluents on the extraction of Zr^4+^ and Hf^4+^ was found to be as follows: carbon tetrachloride > cyclohexane > *n*-hexane, benzene > nitrobenzene > xylene > toluene > chloroform.

The separation factors (SF) between 4f and 5f metals (U/Eu, Th/Eu) as well as for Lu/La, Gd/Eu, and Lu/Eu pairs which demonstrated the 4f-intragroup selectivity, selected as representatives of the beginning, middle, and the end of the 4f-series, are given in Table 3. It is seen that the SFs between Lns and Ans decrease by changing the diluent used in the system in the order CHCl_3_ > [C_1_C_4_im^+^][Tf_2_N^−^] > [C_1_C_10_im^+^][Tf_2_N^−^], whereas their solvent extraction with HPBI molecules increases. This trend concerning selectivity is usually noticed in liquid–liquid extraction chemistry; i.e., the separation becomes poorer when the extractability increases. However, this is not the case obtained for the adjacent lanthanoids Gd/Eu or the middle/heavy pair as Lu/Eu, where the predominance of the ionic environment is remarkable.

**Figure 6:**
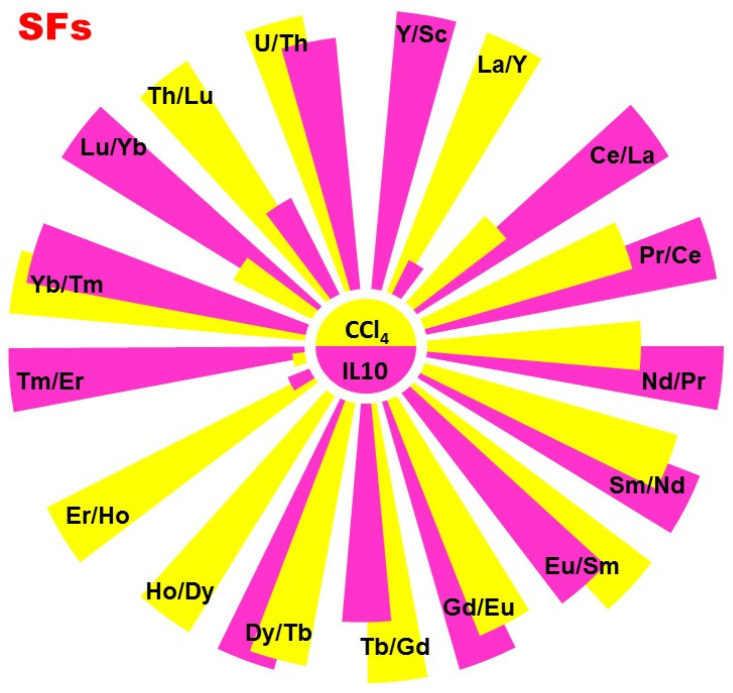
The SFs data visualization of two HPBI solvent systems, including CCl_4_ and [C_1_C_10_im^+^][Tf_2_N^−^] diluents, with a Nightingale rose diagram.

In addition, similar calculations were performed for the selectivity of the two best extraction systems presented in Figure 6. As can be expected, the differences in the obtained SFs values are not significant at all, except maybe for the pairs of metals Ce/La, Er/Ho, and Th/Lu. In general, thorium could be used as a suitable model for some tetravalent actinoids, which are unstable and of course difficult to handle. Importantly, the selectivity of Th^4+^ towards rare earth and transition metal ions has been observed recently to be higher than 1400 times, and the Th/Ni shows a high value of 4520 [61]. The authors investigated a new type of stimulus-responsive IL, [C_20_H_43_Cl_2_NO_4_P]NO_3_, which contains a hydrophobic long alkyl chain, hydrophilic phosphorous-oxygen groups, and a quaternary ammonium cation, which also has pH-responsive properties. This observation shows that even in the presence of transition metal and rare earth ions, this new compound can be used to selectively and efficiently extract the 5f-ions as required (98.7%). The phenanthroline phosphonate extractants have a strong ability to extract trivalent actinoids over lanthanoids from highly acidic HNO_3_ solutions with a good separation ability of An^3+^ over Ln^3+^. Solvent extraction studies carried out by Yang et al. showed that molecules with longer alkyl chains (tetrabutyl-(1,10-phenanthrolin-2,9-diyl) phosphonate) have a stronger extraction ability for U^6+^ and Th^4+^ than its analogue with C2 chains, whereas both ligands exhibit much stronger extraction capacities for Th^4+^ than U^4+^ under the same conditions [65]. On the other hand, the SFs for most of the 4f-pairs can be perceived as relatively equal, for example, the Sm/Nd or Eu/Sm, as shown in Figure 6. Of course, it is not superfluous to emphasize that, even though it is a cheap diluent, tetrachloromethane evaporates quite easily and is not preferred for long-term processes, apart from being environmentally friendly. In other words, the use of more innovative, modern, and green diluents such as ionic liquid compounds would be justified according to the complexity and objectives of metal-selective technology.

### 2.4. EPR Investigation of the Extracting Organic Phases

**Figure 7:**
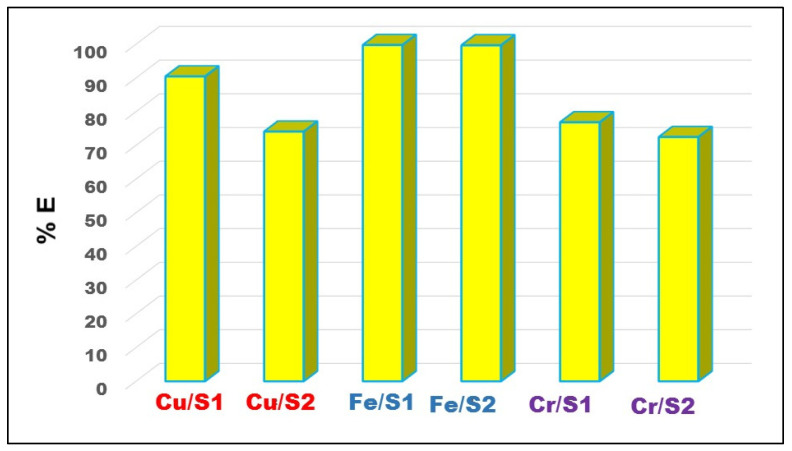
Extraction performance of S1 and S2 ligands ([S1/S2] = 2.5 × 10^−2^ mol/dm^3^) towards Cu^2+^, Fe^3+^, and Cr^3+^ ions (M^n+^]_in_ = 6 × 10^−4^ mol/dm^3^, S1: pH_eq_ = 6.81, S2: pH_eq_ = 6.26). The reported extractability values (%) represent the average of three measurements with deviations of less than 5%.

As a whole, taking into account the good extraction results obtained for copper, iron, and chromium with the two new ligands as presented in Figure 5, additional research has been conducted in order to investigate the corresponding organic phases obtained after solvent extraction using the EPR technique as follows. The extraction abilities of the two molecules were tested for the solvent extraction of these three metal cations alone, as shown in Figure 7. Therefore, the ligand S1 advantage is clearly seen for Cu^2+^, as is the highly efficient process with over 70% for Cr^3+^ and approximately 100% for Fe^3+^ ions.

**Figure 8:**
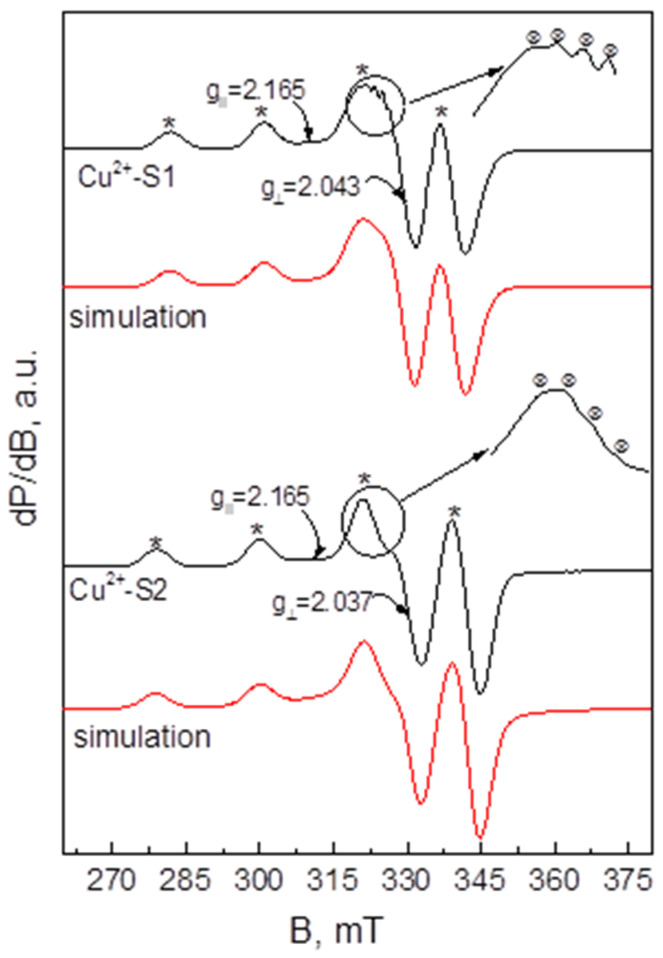
EPR spectra at 120 K of Cu^2+^ complexes with ligands S1 and S2 in an ionic diluent [C_1_C_4_im^+^][Tf_2_N^−^] (black lines) and the corresponding simulated spectra; hyperfine structure lines: [*] and superhyperfine structure lines: [⊗]. The spectra were registered in the frozen solutions.

The EPR measurement of Cu^2+^−S1 and Cu^2+^−S2 complexes in the [C_1_C_4_im^+^][Tf_2_N^−^] environment was conducted in frozen solutions at 120 K. The observed EPR spectra are represented in Figure 8. As can be seen, both spectra consist of parallel and perpendicular components, with g_II_ > g_⊥_ > 2.0023. In the parallel parts, hyperfine structure lines can be observed, indicated with [*]. In addition, five additional lines could be discerned on the third of these lines, ascribed to the presence of the superhyperfine (SHF) structure. The simulation reveals the following EPR parameters that are very close for both the complexes studied—for Cu^2+^−S1: g_II_ = 2.1656, g_⊥_ = 2.0436, and A_II_ = 19.18 mT; for Cu^2+^−S2: g_II_ = 2.1646, g_⊥_ = 2.037, and A_II_ = 21.20 mT. The value of the SHF constant was found to be about 1.5 mT.

For instance, the characteristics of the investigated spectra are related to Cu^2+^ ions in tetragonally distorted octahedral symmetry, as the ground state of Cu^2+^ is the d_x_^2^_-y_^2^ orbital. Taking into account the experimentally evaluated EPR parameters values, a significantly high value of the hyperfine structure constant, A_hfs_, is accompanied by an extremely low value of g_II_. The established relationship is characteristic of Cu^2+^ ions coordinated with four nitrogen atoms in the equatorial plane [66]. In accordance with this conclusion, the lines of the superhyperfine structure result from the interaction of nitrogen nuclear spin (I = 1) and Cu^2+^ electron spin (S = 1/2). In the registered spectra, only part of the SHF splitting lines is distinguishable due to coupling between the S = 1/2 electron spin and four nuclear spins with I = 1.

For the quantitative estimation of Cu^2+^ ion concentration in the studied solutions, EPR software was used. The concentration of Cu^2+^ ions in Cu−S1 was found to be 9.1 × 10^−4^ mol/dm^3^ and of Cu−S2 ca. 1.6 × 10^−3^ mol/dm^3^. In addition, oxime-bridged Cu^2+^ complexes have raised interest as materials with promising magnetic properties in recent years [67].

Furthermore, an EPR experiment was performed on analogous solutions containing Fe^3+^ and Cr^3+^ ions too, but unfortunately, no EPR spectra of paramagnetic species were recorded in these particular cases. Most likely, the concentration of the metal ions in the studied solutions during the solvent extraction process was under the detection limit of the apparatus.

## 3. Experimental Part

### 3.1. Reagents

**Figure 9:**
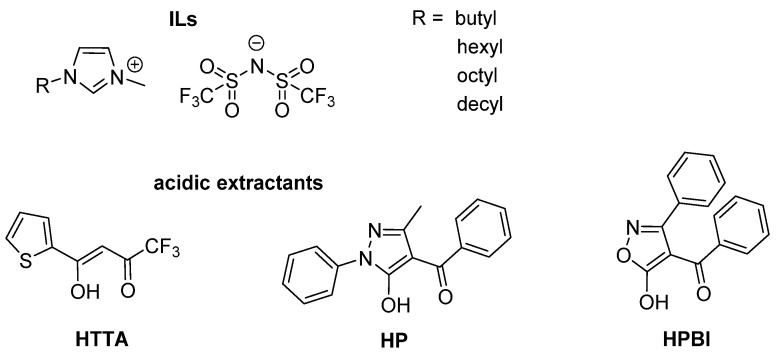
Chemical structures of the chelating ligands and ionic liquids, [C_1_C_n_im^+^][Tf_2_N^−^], (ILs) used in this study.

All reagents were purchased from Aldrich (San Diego, CA, USA), Merck (Darmstadt, Germany), and Fluka (London, UK) and were used without further purification. The three acidic chelating (thenoyltrifluoroacetone, 4-benzoyl-3-methyl-1-phenyl-pyrazol-5-one, and 4-benzoyl-3-phenyl-isoxazol-5-one) extractants (HL) were purchased from Fluka and were used without further purification (purity >98%) (Figure 9). The ionic liquids (1-butyl-, 1-hexyl-, 1-octyl-, and 1-decyl-3-methylimidazolium bis(trifluoromethylsulfonyl)amide) ([C_1_C_n_im^+^][Tf_2_N^−^]), as well as 1-butyl-1-methylpyrrolidinium bis(trifluoromethylsulfonyl)imide ([C_1_C_4_pyr^+^][Tf_2_N^−^]) were supplied by Solvionic (Toulouse, France), all with a purity of 99.5% and an average water content of ca. 200 ppm. The diluents used were CHCl_3_ (Merck, p.a.), C_6_H_6_ (Merck, 99,7%), CCl_4_ (Fluka, p.a.), and C_2_H_4_Cl_2_ (Merck, p.a.). The deuterochloroform is purchased from Deutero GmbH, 99.8 atom% D. The solution of europium ion was prepared from the oxide (Fluka, puriss) by dissolving it in concentrated hydrochloric acid and diluting with distilled water to the required volume. A solution of 0.1 mol/dm^3^ 2-morpholinoethanesulfonic acid (MES) buffer (Alfa Aesar, Ward Hill, MA, USA, 98%) was also applied. ICP MISA Standard 1 (from CPAchem Ltd., Bogomilovo, Bulgaria), Rare earth metals, 18 components were used: 100 mg/L each of Ce, Dy, Er, Eu, Gd, Ho, La, Lu, Nd, Pr, Sc, Sm, Tb, Th, Tm, U, Y, Yb in 5% HNO_3_. All other commercially available analytical grade reagents were used without any further purification.

### 3.2. Synthesis of New Oxime Ligands in Solvent Extraction Chemistry

**Scheme 1:**
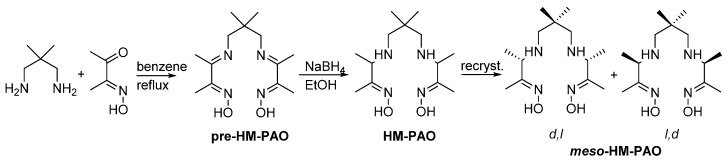
Synthesis of hexamethylpropyleneamine oxime (HM-PAO).

The compound HM-PAO was synthesized from commercially available materials using a known two-step procedure [68] via pre-HM-PAO (Scheme 1). The diastereoisomeric couples *meso* (*d,l* and *l,d*) and *d,l* (*d,d* and *l,l*) were separated via fractional recrystallization [69].

### 3.3. NMR Interaction Measurements

The NMR spectra were recorded on a Bruker Avance II+ 600 spectrometer (Rheinstetten, Germany) at 20 °C. Deuterochloroform (Deutero GmbH) was filtered through a pad of basic alumina prior to use to eliminate the acidic components. The chemical shifts were quoted in ppm in δ-values against tetramethylsilane (TMS) as an internal standard, and the coupling constants were calculated in Hz. The spectra were processed using Topspin 3.1. program.

### 3.4. Solvent Extraction Experiments

Extraction experiments were carried out at room temperature (22 ± 2) °C by mixing the aqueous and organic phases (2 or 1 cm^3^) for 2 h (1500 rpm), which was more than sufficient to attain equilibrium. After 2 min of centrifugation (5000 rpm), the lanthanoid ion concentration in the aqueous phase was determined spectrophotometrically using Arsenazo III (S-20 Spectrophotometer Boeco) [70]. The concentration of the metal ions in the organic phase was determined by the material balance. The extracted solutions were prepared using precisely weighted samples. The acidity of the aqueous phase at equilibrium was measured using a pH meter (pH 211 HANNA, Smithfield, RI, USA) with an accuracy of 0.01 pH unit. The ionic strength was maintained at 0.1 mol/dm^3^ (Na, H)Cl. The initial concentration of the lanthanoid ion (Eu) was 2.5 × 10^−4^ mol/dm^3^ in all experiments.

For competitive extraction tests, a volume of 2 mL of the prepared aqueous solution containing various M^n+^ metal ions (the corresponding nitrate salts were used M^n+^(NO_3_)_n_/M^n+^(NO_3_)_n_·*x*H_2_O) or f-ions mixture was equilibrated for 3 h (1500 rpm) with 2 mL of the organic phase, which included the studied ligand molecule(s). After phase separation, the metal ions concentrations in the aqueous solution were determined by ICP-OES (“Prodigy” High dispersion ICP-OES, Teledyne Leeman Labs, Hudson, NH, USA).

The distribution ratio (*D*) at equilibrium was calculated as follows:

(1) $$D=\frac{{\left[M^{n+}\right]}_{aq,in}-{\left[M^{n+}\right]}_{aq,f}}{{\left[M^{n+}\right]}_{aq,f}}\times \frac{V_{aq}}{V_{IL}}$$

where [*M^n^*^+^]_aq_,_in_ is the concentration of *M^n^*^+^ ion in the aqueous phase before liquid–liquid extraction tests, and [*M^n^*^+^]*_aq_,_f_* is the concentration of the same metal ion in the aqueous phase after extraction. In general, *V_aq_* and *V_IL_* are the volumes of the aqueous and organic phases used to perform experiments, respectively, herein 2:1 (Eu study) or 1:1 *v*/*v* for competitive extraction. For instance, duplicate experiments showed that the reproducibility of *D* measurements was generally within 95%.

Extractability (% E) was evaluated as follows:

(2) $$\mathrm{extractability}=\frac{{\left[M^{n+}\right]}_{aq,in}-{\left[M^{n+}\right]}_{aq,f}}{{\left[M^{n+}\right]}_{aq,in}}\times 100$$

The metal separation between elements in the Periodic table can be estimated using separation factors (SF) determined as the ratio of the distribution ratios of two metal ions, the heavier and lighter ones:

SF = *D*_(Z + n)_/*D*_(Z)_ (3)

### 3.5. EPR Measurements

A Bruker EMX Premium X EPR spectrometer operating in the X-band at 9.4 GHz was used to perform the EPR analysis in the present study. The analysis of the extract solutions was carried out at 120 K by using a thermovariable unit ER 4141. For the experimental calculation of spin concentration, the licensed software “Spin Count” was used, and the spectra simulation was performed with the help of Aniso Spin Fit software. All software utilized was part of Bruker’s Xenon program.

The measurement conditions of the samples were as follows: modulation amplitude, MA = 5, microwave power 0.9039 mW (Att = 21 dB) for Cu^2+^−S1; and modulation amplitude, MA = 10, microwave power 0.9039 mW (Att = 21 dB) for Cu^2+^−S2 complex.

## 4. Conclusions

The synthesis of two new oxime molecules and an assessment of their complexation ability in solution are presented in this study. A new acidic–basic ligand mixture has been introduced to the coordination chemistry in solution during liquid–liquid extraction of Eu^3+^ ions, displaying strong synergism with a β-diketone (HTTA) only. Additionally, the extraction behavior of various s-, p-, d-, and f-metal cations has been investigated using the two oxime molecules. In fact, the S1 molecule seems to be much more effective than oxime S2 in most studied cases, regardless of the type of diluent used. The scientific results indicate that the solvent extraction of rare earth metals with a powerful ligand like HPBI varies with the nature of diluents and follows the order (% E) C_2_H_4_Cl_2_ < CHCl_3_ < C_6_H_6_ < CCl_4_ as well as [C_1_C_4_pyr^+^] < [C_1_C_4_im^+^] < [C_1_C_6_im^+^] < [C_1_C_8_im^+^] < [C_1_C_10_im^+^]. The present study is expected to be helpful in designing synergistic extraction systems for green and sustainable processes, which would extend the application of oxime molecules and ILs in the field.

## Figures and Tables

**Table 1:** Synergistic coefficient obtained for Eu(III) ions in various solvent mixtures.

Solvent Mixture	SC
HPBI + S1	−0.010
HPBI + S2	−0.005
HP + S1	0.029
HP + S2	0.26
HTTA + S1	0.47
HTTA + S2	1.78

**Table 2:** Competitive solvent extraction of rare earths and two 5f-ions with the chelating ligand [HPBI] = 4 × 10^−2^ mol/dm^3^ applying different organic medium at pH = 0.10 in the aqueous phase (V_aq_:V_o_ = 2 mL:2 mL; 0.5 mL 0.1 mol/dm^3^ MES). The reported extractability values (%) represent the average of three measurements with deviations of less than 5%.

Diluent	Sc	Y	La	Ce	Pr	Nd	Sm	Eu	Gd	Tb	Dy	Ho	Er	Tm	Yb	Lu	U	Th
**M^n+^ _in_**	**13.01 ± 0.44**	**12.91 ± 0.22**	**12.65 ± 0.22**	**12.86 ± 0.26**	**12.79 ± 0.32**	**13.23 ± 0.26**	**12.94 ± 0.22**	**13.05 ± 0.22**	**13.30 ± 0.40**	**13.07 ± 0.26**	**12.82 ± 0.34**	**13.14 ± 0.35**	**12.67 ± 0.20**	**13.01 ± 0.21**	**13.04 ± 0.40**	**12.95 ± 0.30**	**13.08 ± 0.28**	**12.95 ± 0.60**
**CHCl_3_**	65.2%	1.6%	−	−	−	1.9%	2.6%	3.7%	−	1.8%	−	3.8%	8.9%	6.8%	3.3%	2.5%	3.5%	73.3%
**CCl_4_**	100%	59.5%	18.8%	16.2%	18.6%	29.8%	54.8%	64.2%	68.4%	76.1%	79%	78.2%	99.2%	78.7%	76%	69.7%	50.8%	80.2%
**C_6_H_6_**	96.2%	3.7%	−	−	0.7%	2.0%	3.8%	4.7%	3.2%	4.2%	8.2%	6.2%	12.4%	9.3%	4.8%	5.7%	6.4%	76.3%
**C_2_H_4_Cl_2_**	53.9%	−	−	−	−	−	0.7%	0.6%	−	−	−		5.7%	3.8%	−	1.5%	1%	75%
**[C_1_C_4_im^+^]**	78%	22.1%	−	−	−	6.6%	20.7%	26.9%	28.1%	34.3%	40.7%	42.2%	62.6%	59.5%	69%	64%	23%	75%
**[C_1_C_6_im^+^]**	78.7%	39.5%	−	−	3.4%	13.45%	34.6%	40%	47.6%	53.3%	58.8%	59.9%	81.8%	71.4%	60.9%	73.4%	34%	77.4%
**[C_1_C_8_im^+^]**	79.4%	47.5%	−	−	6.7%	15.6%	40.8%	48.6%	56.8%	60.9%	66.7%	67.4%	91.5%	79.4%	68%	77.8%	39%	77.3%
**[C_1_C_10_im^+^]**	81.7%	54%	5.0%	7.7%	11.3%	23.5%	49.3%	56.4%	62.9%	67.6%	71.9%	71.7%	95.4%	75.2%	71.2%	81.2%	46.2%	78.9%
**[C_1_C_4_pyr^+^]**	85.4%	19.82%	−	−	3.0%	10.3%	22.4%	22.9%	25.5%	31.8%	36.5%	35.8%	54.6%	43.5%	33.8%	51.5%	22.2%	77.6%

**Table 3:** Selectivity between f-elements.

Diluent	Lu/Eu	U/Eu	Th/Eu	Gd/Eu	Lu/La
CHCl_3_	0.64	0.93	70.58	0.37	
[C_1_C_4_im^+^][Tf_2_N^−^]	4.82	0.81	8.18	1.06	
[C_1_C_10_im^+^][Tf_2_N^−^]	3.33	0.66	2.89	1.31	80.83

## Data Availability

Data sharing is not applicable to this article.

## Supplementary Materials

The following supporting information can be downloaded at: https://www.mdpi.com/article/10.3390/molecules28227467/s1, Figure S1. Isoxazolone signals in 1H NMR spectra of isoxazolone, isoxazolone:pre-HMPAO 1:1 mixture, and isoxazolone:HM-PAO 1:1 mixture; Figure S2. Extractability of La(III), Eu(III) and Lu(III) ions ([Ln^3+^] = 0.01 mM) by 1 mM pre-HM-PAO in [C_1_C_4_im^+^][Tf_2_N^−^] medium. Figure S3. Log*D* vs. pH for Ln^3+^ extraction by [HPBI] = 1 mM alone and the corresponding mixtures [S1] = [S2] = 1 mM in [C_1_C_4_im^+^][Tf_2_N^−^] medium: L –HPBI, S1—pre-HM-PAO, S2—HM-PAO. Figure S4. Investigation of pH_in_ ~2.50 (in all cases) vs. pH_eq_ without metal in the system: 1−pH_in_; 2−HTTA+S1 and HTTA+S2; 3−HPBI+S1 and HPBI+S2; 4−HP+S1 and HP+S2; 5−S1 and S2 at [L] = 1 × 10^−2^ M; 6− S1 and S2 at [L] = 5 × 10^−3^ mol/dm^3^; 7−S1 and S2 at [L] = 2 × 10^−2^ mol/dm^3^. Figure S5. Log*D*_T,S_ vs. log[HTTA] for Eu^3+^ ([Eu^3+^]_in_ = 2.5 × 10^−4^ mol/dm^3^) synergistic solvent extraction with [S2] = 3 × 10^−3^ mol/dm^3^ (V_o_/V_aq_ = 2 mL: 2 mL, 0.1 mol/dm^3^ NaCl). Table S1. Competitive solvent extraction of rare earths and two 5f-ions with the chelating ligand [HTTA] = 3 × 10^−2^ mol/dm^3^ or [S1/S2] = 8 × 10^−3^ mol/dm^3^ alone as well as in combinations applying CHCl_3_ (lilac color) or [C_1_C_6_im^+^][Tf_2_N^−^] (in red) at pH < 0.10 of the aqueous phase (V_aq_:V_o_ = 2 mL:2 mL; 0.5 mL 0.1 mol/dm^3^ MES). The reported extractability values (%) represent the average of three measurements with deviations of less than 5%.
