# Supplementary material for: New Sustainable Solvent Extraction Pathways for Rare Earth Metals via Oximes Molecules
Source: Molecules. 2023 Nov 7;28(22):7467. doi: 10.3390/molecules28227467 (PMC10673299; doi:10.3390/molecules28227467)
Supplement: Supplementary file 1 [file molecules-28-07467-s001.zip › molecules-2694845-supplementary.pdf]

Supplementary Material

NEW SUSTAINABLE SOLVENT EXTRACTION PATHWAYS FOR RARE EARTH METALS VIA OXIME MOLECULES

Maria Atanassova[a*], Rositsa Kukeva[b] and Vanya Kurteva[c]

[a]University of Chemical Technology and Metallurgy,
Department of General and Inorganic Chemistry, 8 Kliment Okhridski blvd., 1756 Sofia, Bulgaria
E-mail: ma@uctm.edu
[b]Institute of General and Inorganic Chemistry,
Bulgarian Academy of Sciences, Acad. G. Bonchev street, Block 11, 1113 Sofia, Bulgaria
[c]Institute of Organic Chemistry with Centre of Phytochemistry,
Bulgarian Academy of Sciences, Acad. G. Bonchev street, Block 9, BG 1113, Sofia, Bulgaria

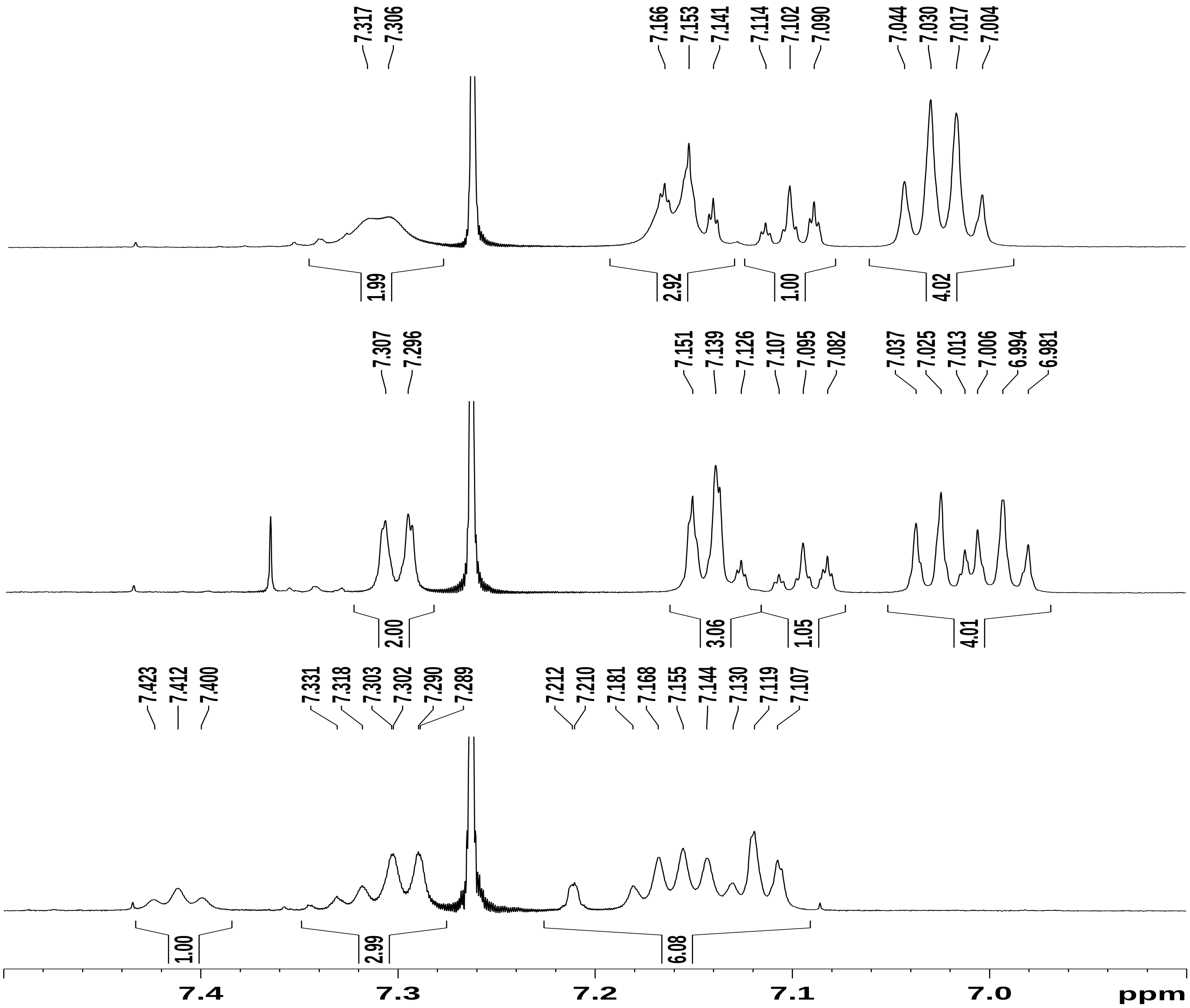

Figure S1. Isoxazolone signals in ^{1}H NMR spectra of isoxazolone (bottom), isoxazolone:pre-HMPAO 1:1 mixture (middle), and isoxazolone:HM-PAO 1:1 mixture (top).

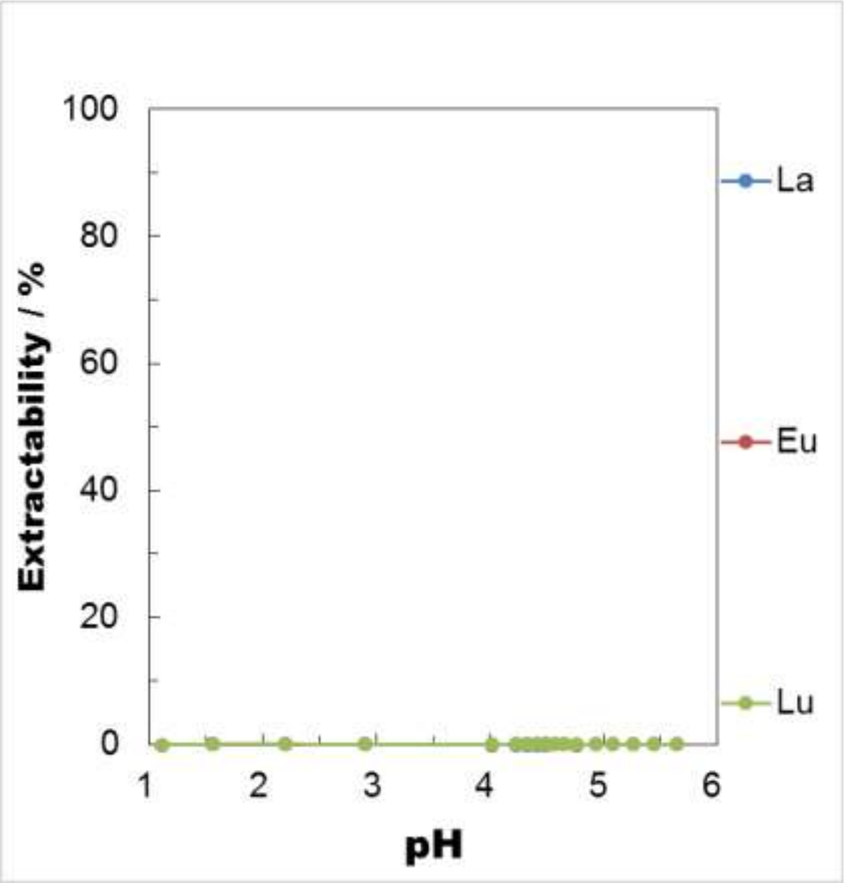

Figure S2. Extractability of La(III), Eu(III) and Lu(III) ions ([Ln^{3+}]=0.01 mM) by 1 mM pre-HM-PAO in [$C_1C_4im^+$][Tf_2N^-] medium.

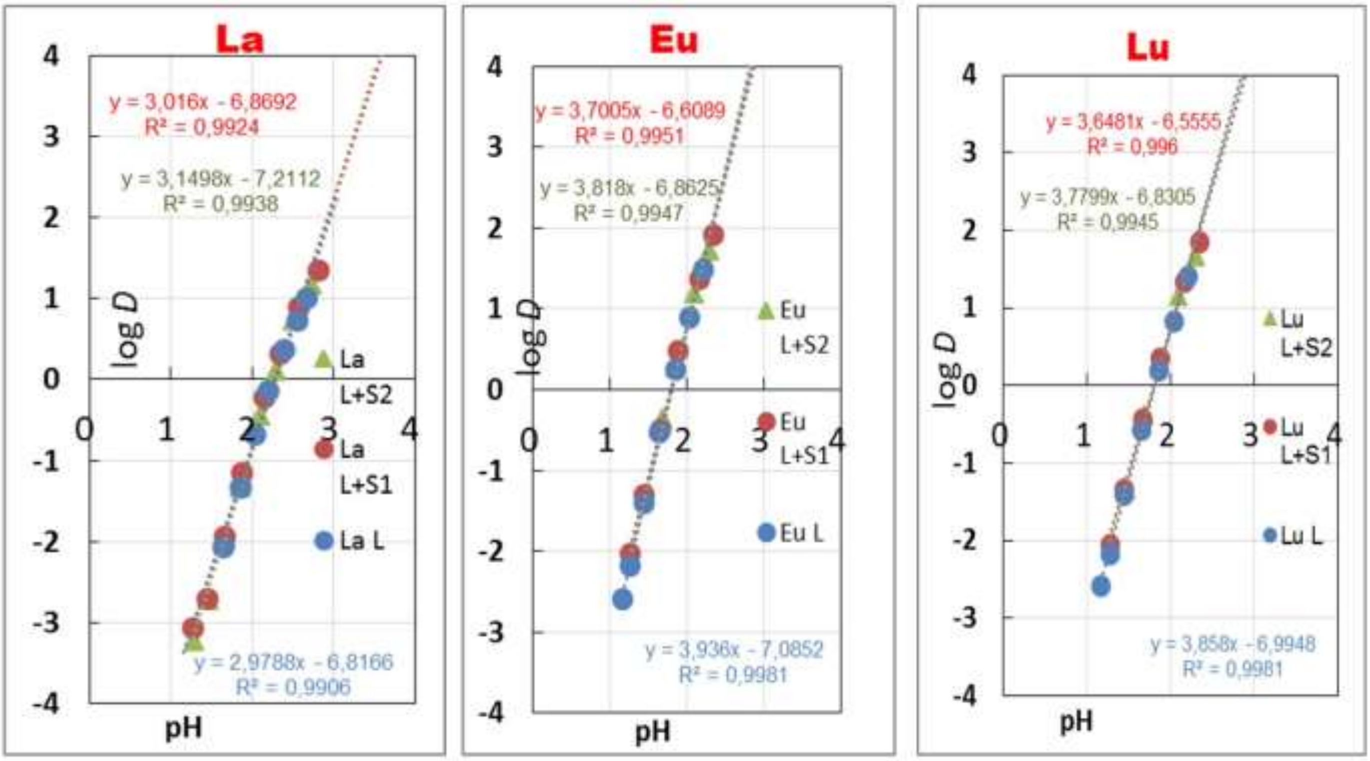

Figure S3. Log*D* *vs.* pH for Ln^{3+} extraction by [HPBI] = 1 mM alone and the corresponding mixtures [S1] = [S2] = 1 mM in [$C_1C_4im^+$][Tf_2N^-] medium: L –HPBI, S1 – pre-HM-PAO, S2 – HM-PAO.

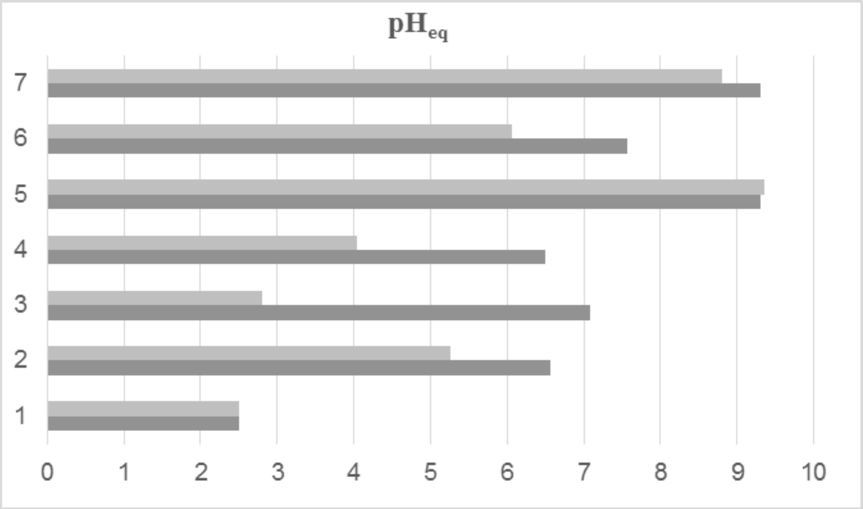

Figure S4. Investigation of pH_{in} ~2.50 (in all cases) *vs.* pH_{eq} without metal in the system:

1−pH_{in}; 2−HTTA+S1 and HTTA+S2; 3−HPBI+S1 and HPBI+S2; 4−HP+S1 and HP+S2; 5−S1 and S2 at [L]=1×10^{-2} M; 6− S1 and S2 at [L]=5×10^{-3} mol/dm^3 ; 7−S1 and S2 at [L]=2×10^{-2} mol/dm^3.

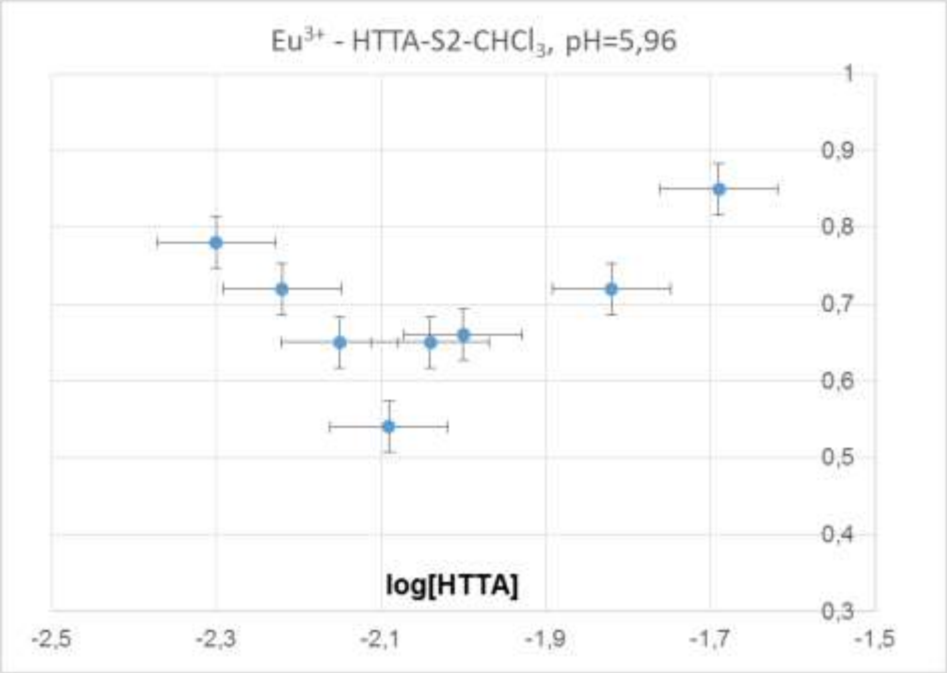

Figure S5. Log$D_{T,S}$ *vs.* log[HTTA] for Eu^{3+} ([Eu^{3+}]$_{in}$= 2.5×10^{-4} mol/dm^3) synergistic solvent extraction with [S2]=3×10^{-3} mol/dm^3 (V_o/V_{aq}=2 ml: 2 ml, 0.1 mol/dm^3 NaCl).

Table S1. Competitive solvent extraction of rare earths and two 5f-ions with the chelating ligand [HTTA]=3×10^{-2} mol/dm^3 or [S1/S2]= 8×10^{-3} mol/dm^3 alone as well as in combinations applying $CHCl_3$ (lilac color) or $[C_1C_6im^+][Tf_2N^-]$ (in red) at pH<0.10 of the aqueous phase (V_{aq}:V_o=2 ml : 2 ml; 0.5 ml 0.1 mol/dm^3 MES). The reported extractability values (%) represent the average of three measurements with deviation less than 5%.

M^{n+}	0	S1	S2	HTTA	HTTA+S1	HTTA+S2	S1	S2	HTTA	HTTA+S1	HTTA+S2
	mg/l										
Ce	20.22±0.22	20.56±0.44	22.21±0.26	20.09±0.19	21.21±0.34	21.26±0.20	21.75±0.18	21.31±0.34	21.52±0.22	22.17±0.22	22.43±0.10
Dy	18.93±0.32	19.44±0.34	20.93±0.42	19.47±0.21	19.89±0.22	19.47±0.24	20.05±0.12	19.52±0.26	19.84±0.24	20.54±0.38	20.78±0.34
Er	15.76±0.16	15.34±0.12	15.22±0.98	14.93±0.40	14.92±0.24	15.05±0.32	14.78±0.19	14.69±0.11	14.52±0.42	14.25±0.52	14.47±0.26
Eu	11.94±0.09	11.86±0.05	11.75±0.05	11.89±0.05	11.90±0.12	11.94±0.05	11.93±0.10	12.01±0.10	12.00±0.09	11.98±0.08	11.98±0.08
Gd	23.16±0.42	24.15±0.62	26.92±0.40	25.73±0.60	26.61±0.42	26.36±0.62	27.42±0.30	27.04±0.60	27.77±0.30	28.81±0.42	29.34±0.30
Ho	21.47±0.62	22.67±0.60	22.66±0.50	23.69±059	24.45±0.40	24.15±0.44	25.11±0.40	24.56±0.40	25.19±0.34	26.13±0.46	26.60±0.14
La	19.23±0.31	19.52±0.44	20.88±0.09	19.92±0.28	20.22±0.40	19.73±0.26	20.17±0.14	19.72±0.26	19.90±0.26	20.41±0.34	20.62±0.14
Lu	19.11±0.24	19.51±.30	20.95±0.18	19.69±0.30	20.07±0.24	19.65±0.22	20.03±0.12	19.55±019	19.79±0.18	20.35±0.20	20.52±0.09
Nd	19.71±0.26	20.32±0.40	21.82±0.26	20.70±0.22	21.32±0.30	20.92±0.34	21.50±0.18	21.04±0.27	21.31±0.20	21.93±0.09	22.20±0.14
Pr	16.17±0.22	16.24±0.48	16.90±0.32	15.47±0.41	15.53±0.30	14.95±0.10	14.99±0.10	14.53±0.28	14.27±0.19	14.40±0.08	14.34±0.20
Sc	20.21±0.50	21.26±0.43	25.55±0.14	21.96±0.24	22.53±0.18	22.37±0.24	22.82±0.10	22.53±0.14	22.87±0.18	23.34±0.24	23.57±0.20
Sm	18.89±0.20	19.36±0.20	20.60±0.10	19.42±0.21	19.78±0.20	19.38±018	19.87±0.11	19.40±0.31	19.55±0.26	20.06±0.08	20.23±0.08
Tb	22.78±0.63	24.32±0.52	27.08±0.52	25.84±0.48	26.71±0.60	26.62±0.48	27.72±0.44	27.63±0.42	27.98±0.41	29.17±0.48	29.92±0.40
Th	20.49±0.44	21.46±0.24	21.46±.24	22.14±0.26	22.81±0.32	22.52±0.30	23.20±0.11	22.77±0.12	23.22±0.22	23.99±0.10	24.35±0.10
Tm	20.12±0.20	20.76±0.41	20.76±0.46	21.26±0.28	21.79±0.36	21.46±0.34	22.11±0.20	21.58±0.28	21.99±0.36	22.76±040	23.10±0.40
U	20.42±0.26	21.24±0.40	21.24±0.40	20.05±0.26	22.69±0.36	22.38±0.30	22.99±0.16	22.60±0.50	22.96±0.34	23.76±0.09	24.11±0.11
Y	23.24±0.86	24.76±0.52	24.76±0.52	26.78±0.44	27.79±0.46	28.04±0.60	28.90±0.19	29.09±0.42	29.80±0.20	30.63±0.42	31.19±0.34
Yb	20.10±0.68	21.28±0.76	21.28±0.76	22.62±0.43	23.41±0.42	23.25±0.46	24.06±0.24	23.69±0.34	24.18±0.10	25.04±0.12	25.40±0.24
